# Experimental Investigation on the Joining of Aluminum Alloy Sheets Using Improved Clinching Process

**DOI:** 10.3390/ma10080887

**Published:** 2017-08-01

**Authors:** Chao Chen, Shengdun Zhao, Xiaolan Han, Xuzhe Zhao, Tohru Ishida

**Affiliations:** 1 School of Mechanical Engineering, Xi’an Jiaotong University, Xi’an 710049, China; 2 Graduate School of Technology, Industrial and Social Sciences, Tokushima University, Tokushima 770-8506, Japan; materialusing3@163.com; 3 Mechanical Engineering College, Xi’an Shiyou University, Xi’an 710049, China; hanxiaolan007@163.com; 4 School of Engineering Technology, Purdue University, West Lafayette, IN 47906, USA; longlong123sci@163.com

**Keywords:** aluminum alloy, improved clinching, material flow, geometrical parameters, failure mode, strength

## Abstract

Aluminum alloy sheets have been widely used to build the thin-walled structures by mechanical clinching technology in recent years. However, there is an exterior protrusion located on the lower sheet and a pit on the upper sheet, which may restrict the application of the clinching technology in visible areas. In the present study, an improved clinched joint used to join aluminum alloy sheets was investigated by experimental method. The improved clinching process used for joining aluminum alloy evolves through four phases: (a) localized deformation; (b) drawing; (c) backward extrusion; and (d) mechanical interlock forming. A flat surface can be produced using the improved clinching process. Shearing strength, tensile strength, material flow, main geometrical parameters, and failure mode of the improved clinched joint were investigated. The sheet material was compressed to flow radially and upward using a punch, which generated a mechanical interlock by producing severe localized plastic deformation. The neck thickness and interlock of the improved clinched joint were increased by increasing the forming force, which also contributed to increase the strength of the clinched joint. The improved clinched joint can get high shearing strength and tensile strength. Three main failure modes were observed in the failure process, which were neck fracture mode, button separation mode, and mixed failure mode. The improved clinched joint has better joining quality to join aluminum alloy sheets on the thin-walled structures.

## 1. Introduction

In recent years, lightweight materials have been widely used to build the thin-walled structures [1]. One of the most widely used lightweight materials is aluminum alloy, which has good forming properties [2]. Welding technology is often used for joins in traditional building structures. However, the oxidation layer on the surface and high thermal conductivity will make it difficult to join aluminum alloy using welding technology [3].

Self-piercing riveting [4], mechanical clinching [5,6,7,8], hole joining and other technologies [9,10,11,12] are better choices to join lightweight materials. The self-pierce riveted joint can get a higher static strength with the help of the rivet [13]. However, the rivet will impale the upper sheet, which may generate damage on the sheet. Mucha et al. investigated the mechanical clinch-riveting process using a solid rivet [14,15]. The upper sheet is not impaled and damaged in the mechanical clinch-riveting process [16]. However, the rivet used in the riveted joint may increase the cost and weight. In order to build the lightweight thin-walled structures, it is better to use the joint without the rivet [17]. 

Another effective mechanical joining technology is mechanical clinching [18,19,20]. In recent years, mechanical clinching is widely used in engineering structures and components. No rivet is needed in the clinching process. A mechanical interlock is produced to hook the sheets together by severe plastic deformation in the clinching process [21,22]. The clinching tools, energy absorption, static strength, fatigue strength, geometrical parameters, and process parameters have been investigated by many researchers [23,24,25,26,27,28]. Mechanical clinching is suitable to join many different materials, such as steel, aluminum, copper, magnesium titanium, Carbon Fiber Reinforced Polymer, Glass Fiber Reinforced Polymer, and other polymers [29,30,31,32,33,34,35,36,37,38]. One of the main limitations for the mechanical clinched joint is represented by the stress concentration which may result in the cracks in the sheets with reduced ductility [39,40,41]. In order to avoid the cracks, a pre-heating process is always combined with the clinching process [42]. Another method is to use the rotating tools, which are also effective to improve the material flow [43]. However, there is a high protrusion generated on the lower sheet and a deep pit generated on the upper sheet in the mechanical clinching process. The high protrusion and deep pit will limit the application of the clinched joint in the visible areas.

Many researchers explored and investigated new joining processes to avoid the exterior protrusion. In order to achieve a lower protrusion, Chen et al. [44] explored a new method to compress the exterior protrusion. In his study, the protrusion was reshaped by flat dies to avoid the exterior protrusion with a rivet. However, the cost and weight for building the thin-walled structures were increased by the additional rivet. Chen et al. [45] also proposed another method using no rivet to avoid the increase of the cost and weight. However, while the weight and cost were reduced, the strength was also reduced using this new method. 

Wen et al. [46] investigated a hole-clinching technology to produce the clinched joint. A cylindrical punch was used to embed upper sheet into the hole, forming a mechanical interlock to join the sheets together. A hole must be produced on the lower sheet before the flat hole-clinching process, which may also affect the processing efficiency. Neugebauer et al. [47] discussed the developing trend of dieless clinching technology. There is still a protrusion generated on the surface of the sheet, but this protrusion is lower than the conventional clinched joint.

In order to expand the application range of the clinching technology, an exterior protrusion should be avoided on the lower sheet. Gerstmann and Awiszus [48] discussed a new mechanical clinching technology which can produce clinched joint with a flat surface. Lüder et al. [49] also carried out some experiments on joining quality of the joint using wood materials and aluminum. There are many papers related to the clinched joint with a high protrusion, but few about the clinched joint with a lower protrusion. It is important to carry out a comprehensive study on joining quality of the improved clinched joint.

In the present study, an improved clinching process was investigated by experimental methods. Al1060 sheet with a thickness of 2 mm was used to carry out the experiments. Different forming forces were used to produce different improved clinched joints. Shearing strength, tensile strength, material flow, main geometrical parameters, and failure mode of the improved clinched joint were investigated. The improved clinched joint was proved to be efficient and feasible for joining Al1060 sheet.

## 2. Principle of the Improved Clinching Process 

**Figure 1:**
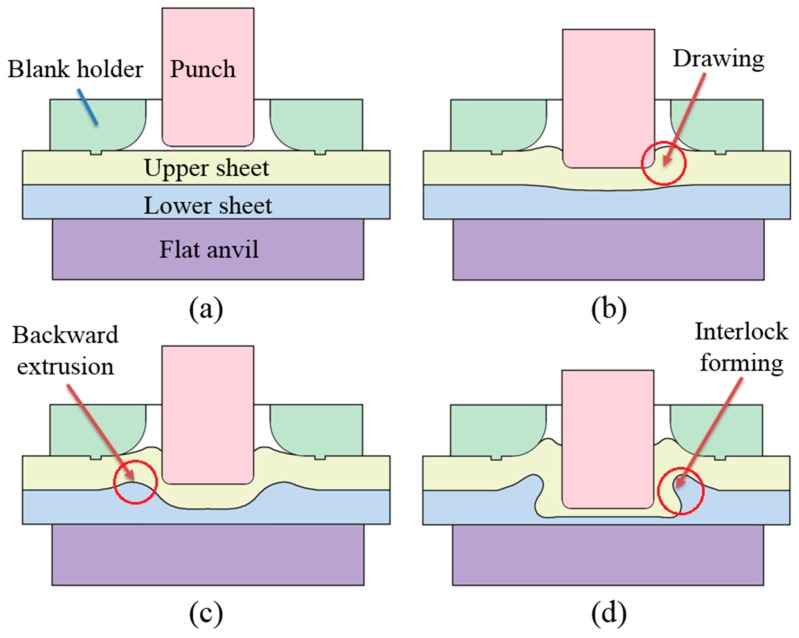
Improved clinching process: (**a**) localized deformation; (**b**) drawing; (**c**) backward extrusion and (**d**) interlock forming.

The improved clinching process is intended to generate a mechanical interlock by generating plastic deformation. The improved clinching process evolves through four phases as shown in Figure 1, which are: (a) localized deformation; (b) drawing; (c) backward extrusion; and (d) mechanical interlock forming. 

Initially, the sheets are placed on the flat anvil. Then the punch moves to push the material to flow downward and radially. With the constraint of the blank holder, the material of the upper sheet which flows radially will move upward. The material which moves opposite to the movement of the punch will be gathered between the punch and blank holder. In the third phase, the material of the lower sheet is also compressed to move opposite to the movement of the punch. During the last phase, all of the sheets are deformed plastically, which generates a mechanical interlock to hook the sheets together. With the help of the flat anvil, there is no exterior protrusion generated on the lower sheet.

**Figure 2:**
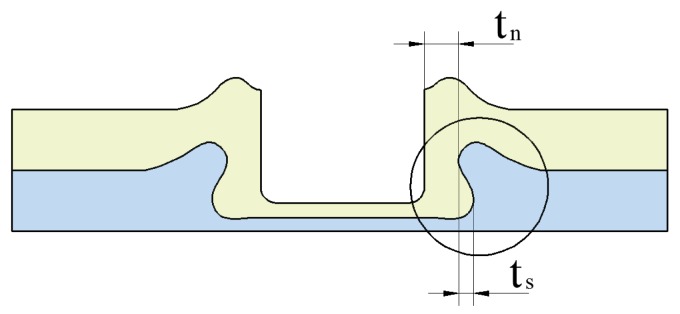
Main geometrical parameters of the improved clinched joint.

The improved clinched joint is produced using mechanical processes. Thus, the joining quality of the improved clinched joint mainly depends on the geometrical parameters of the joint profile other than the process-induced strain hardening and mechanical properties of the material [50]. As shown in Figure 2, the main geometrical parameters include the interlock (*t_s_*) and neck thickness (*t_n_*).

## 3. Materials and Experimental Procedure

### 3.1. Materials

In this study, Al1060 was chosen to carry out the improved clinching experiments. In order to ensure the accuracy of the experimental results, all of the Al1060 sheets were cut from a large rolled Al1060 plate. The thickness of the Al1060 sheet is 2.0 mm, length is 80 mm, and width is 25 mm. The mechanical properties of Al1060 were tested by Instron 5982 testing machine (Instron Company, Grove City, PA, USA). According to the test results, the elastic modulus of Al1060 is 54.5 GPa, tensile strength is 117.9 MPa, and poisson’s ratio is 0.33.

### 3.2. Mechanical Improved Clinching Process 

**Figure 3:**
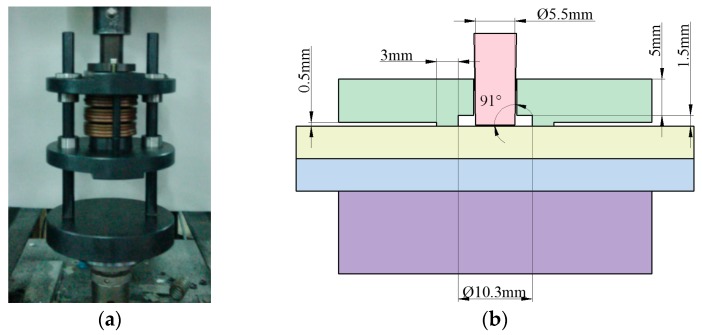
Improved clinching tools: (**a**) dies; (**b**) main geometrical parameters.

**Figure 4:**
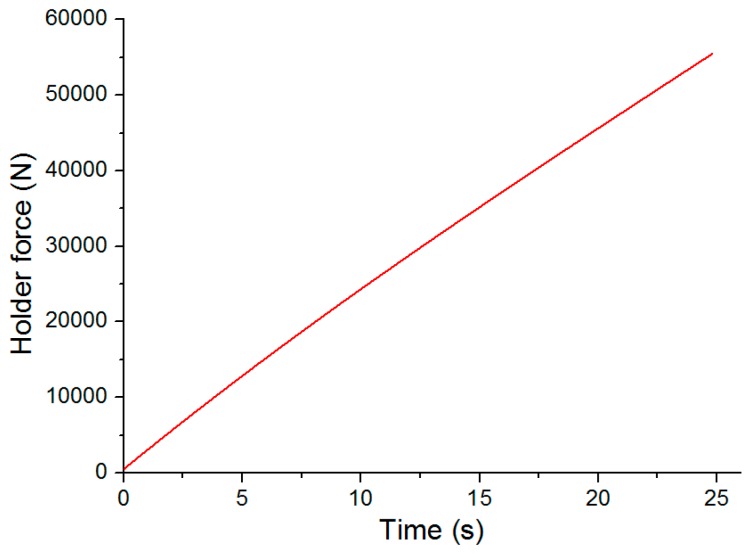
Holder force.

The mechanical improved clinching process was also carried out on the Instron 5982 machine (Instron Company, Grove City, PA, USA). The clinching tools for the improved clinched joint are shown in Figure 3. The main clinching tools include a punch, a flat anvil, and a blank holder. Different forming forces such as 40, 50, 60, 70, 80, and 90 kN were applied in the improved clinching process to produce different clinched joints. The speed of the punch in the clinching process was 0.5 mm/s. The blank holder was connected to the punch by disc springs. Disc springs were also used to generate holder force on the blank holder. The holder force on the blank holder was shown in Figure 4. The preload of the blank holder was set to 500 N.

### 3.3. Cross Sections 

The improved clinched joints were produced in the mechanical improved clinching process with different forming forces. The Al1060 sheets were hooked together by the mechanical interlock, thus the geometrical parameters are essential research contents in this study [51]. The improved clinched joint was cut by wire-cut electrical discharge machining technology along the center line of the joint to get the cross-sectional profile. The cross-section of the improved clinched joint was observed by a metallurgical microscopy produced by Nikon (Tokyo, Japan). According to the cross-sectional profile of the improved clinched joint, the main geometrical parameters can be measured and recorded. 

### 3.4. Static Strength Test

The shearing test and tensile test are often used to ensure joining safety. The maximum strength can be measured in these tests using the Instron 5982 machine (Instron Company, Grove City, PA, USA). The speed for the static shearing and tensile tests was 2 mm/min. Five specimens were tested to calculate the average strength for each forming condition.

**Figure 5:**
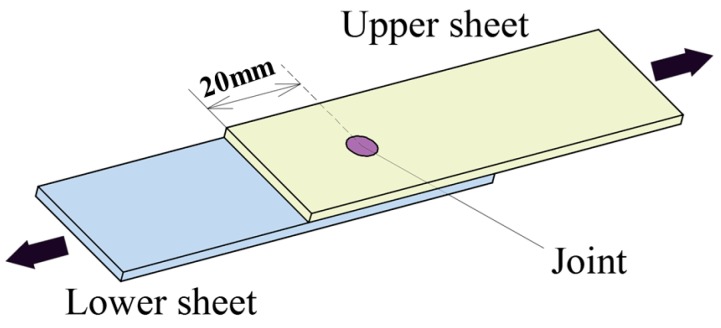
Specimen used for shearing test.

As shown in Figure 5, a specimen used for shearing test was placed as lap-shearing type [52]. The improved clinched joint was located on the center of the metal sheets. The lower sheet was fixed by the fixture, and the upper sheet was grasped by another fixture to move upward until the improved clinched joint was failed. 

**Figure 6:**
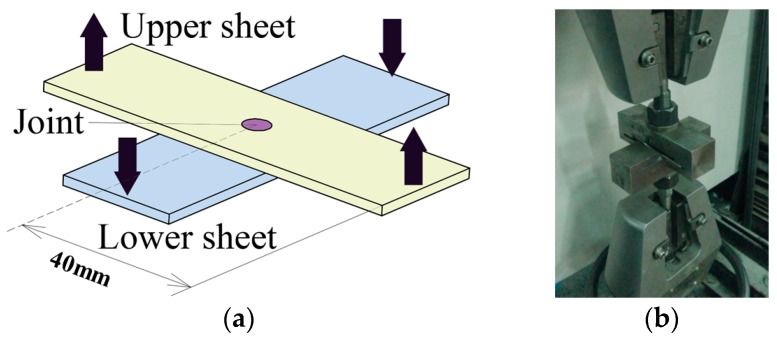
Specimen used for tensile test: (**a**) Specimen; (**b**) tensile testing fixtures.

As shown in Figure 6a, the specimen used for the tensile test was placed as a cross-shaped type. The improved clinched joint was also located on the center of the metal sheets. A pair of special tensile testing fixtures was designed to measure the tensile strength, as shown in Figure 6b. The lower sheet was fixed by the lower fixture, and the upper sheet was pulled by the upper fixture to move upward until the joint was failed.

## 4. Results and Discussion

### 4.1. Material Flow

**Figure 7:**
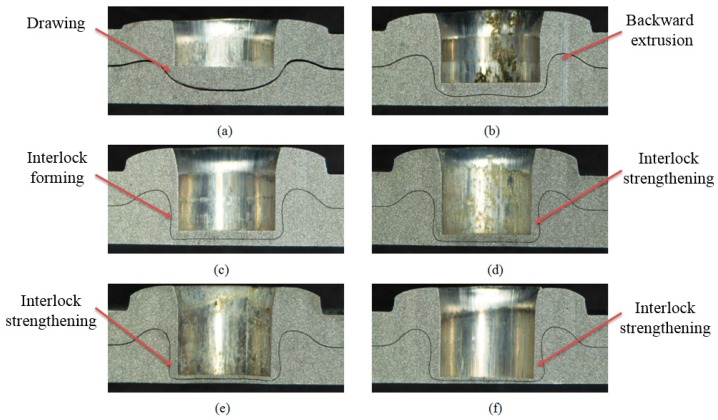
Cross-sectional profiles of joints formed by different forming forces: (**a**) *F* = 40 kN; (**b**) *F*= 50 kN; (**c**) *F* = 60 kN; (**d**) *F* = 70 kN; (**e**) *F* = 80 kN; (**f**) *F* = 90 kN.

The cross-sectional profile of the improved clinched joint is directly influenced by the evolution of material flow during the mechanical improved clinching process [53]. A series of cross-sectional profiles of improved clinched joints is shown in Figure 7 to display material flow for forming an improved clinched joint.

For the improved clinched joints with forming forces of 40 and 50 kN, there is no mechanical interlock generated. The sheets are not joined together without an interlock. For the improved clinched joints with forming forces between 60 and 90 kN, a mechanical interlock was formed because of the plastic deformation. The two Al1060 sheets are clinched together by the interlock which is generated by the material flowing opposite to the movement of the punch. 

The Al1060 sheets were compressed and deformed by the cylindrical punch. The flat anvil could restrain the downward flow of the material, which leaded the material flow radially. With the clinching tools to stamp the sheets in place, radial flow of the material was prevented. Then the material flowed opposite to the movement of the punch and moved into the space between the blank holder and punch. With the severe radial and upward material flow of the lower sheet, a reliable interlock was formed plastically. Then the two sheets were joined together by the mechanical interlock. With the flat anvil to restrain the material flow downward, the lower sheet contacting the flat anvil was completely flat with no exterior protrusion. Compared with conventional clinching process, a higher forming force is required in the flat clinching process.

During the conventional clinching process, the sheet material needs to flow downward and radially to fill the die-sided cavity. Unlike the conventional clinching process, the material of the sheets needs to flow upward and radially to form the mechanical interlock in the improved clinching process. 

During the conventional clinching process, the blank holder is used to keep and clamp the sheets on the sliding sectors. During the improved clinching process, the blank holder is used to restrain the horizontal flow of materials and ensures the material flows opposite to the movement of the punch.

### 4.2. Main Geometrical Parameters

There is no thermal reaction or chemical reaction produced in the improved clinching process. The aluminum alloy sheets are joined and clinched together by a mechanical interlock, thus the geometrical parameters of the joint profile can influence the joining quality of the improved clinched joint [54]. 

When the forming force was set to 40 or 50 kN, there was no mechanical interlock generated between the sheets, and the improved clinched joint was not produced. Thus, only the improved clinched joints with different forming forces of 60, 70, 80, and 90 kN are considered in the following study.

**Figure 8:**
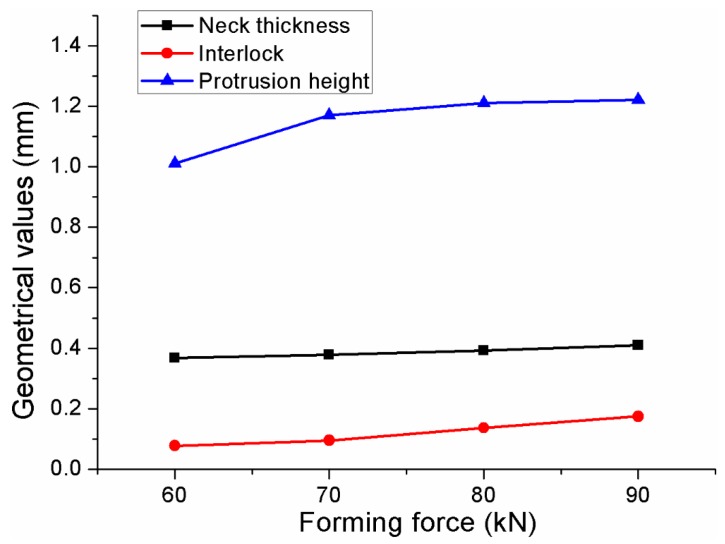
Main geometrical parameters of the improved clinched joints.

Interlock and neck thickness are two pivotal geometrical parameters for the improved clinched joint in this study [55]. The main geometrical parameters of the improved clinched joints are shown in Figure 8. The neck thickness and interlock were increased with the forming force, which was generated by the radial and upward movement of the materials. One part material of the upper sheet was compressed to flow radially to be implanted into the lower sheet, which generates the mechanical interlock with severe plastic deformation. 

A flat surface was produced on the lower sheet, and a protrusion was produced on the upper sheet in the improved clinching process. As shown in Figure 8, the protrusion height was increased with the forming force. For the conventional clinched joint, there is a pit on the upper sheet and a protrusion on the lower sheet. So the conventional clinched joint can’t be used in the visible areas or functional areas. The pit and the protrusion also have an adverse effect on the subsequent processing such as assembly and so on. However, in the improving clinching process, there is a flat surface produced on the lower sheet, which makes it suitable to be used in visible areas or functional areas. Though the protrusion was compensate in the upper sheet, one flat surface is enough to be used in the visible areas or functional areas. Compared with the conventional clinched joint, the improved clinched joint has a broader range of applications.

**Figure 9:**
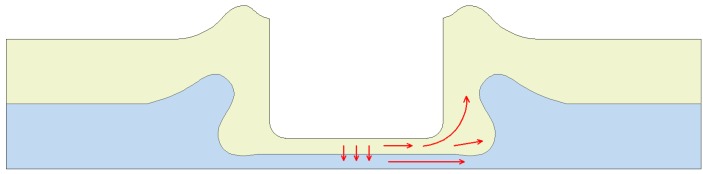
Material flow to increase the interlock and neck thickness.

As shown in Figure 9, the material flow to increase the interlock and neck thickness is indicated by the red narrow. In the clinching process, the sheets were compressed by the upper punch. The bottom thickness was reduced with the movement of the punch, then the material of the bottom part was compressed to flow radially. With the blank holder to fix the sheets, the material flowing radially could be gathered in the interlock area. With the increase of the forming force, more and more materials would flow radially to reinforce the mechanical interlock. Thus, the interlock was increased with the forming force. In addition, with the blank holder to fix the sheets, radial flow of the material was also prevented. Then the material flowed opposite to the movement of the punch and moved into the space between the blank holder and punch, which increased the neck thickness.

### 4.3. Failure Mode

There are three main failure modes in the process of failure: neck fracture mode, button separation mode, and mixed failure mode [56,57]. When the upper sheet is separated from the other sheet, this failure mode is called button separation. When the neck is fractured in the process of failure, the failure mode is called neck fracture. Mixed failure mode is a combination of neck fracture mode and button separation mode. The fracture modes of the improved clinched joint are similar with the conventional clinched joint. 

**Figure 10:**
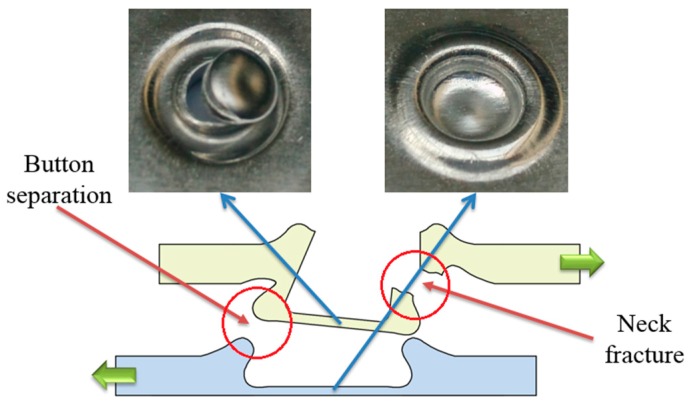
Mixed failure mode in the shearing test.

During the shearing strength test, mixed failure mode and neck fracture mode were observed in this study. As shown in Figure 10, the improved clinched joint formed by a force of 60 kN was failed as the mixed failure mode during the shearing test. With the mechanical interlock to hook the sheets together, the shearing force was mainly exerted on neck of the improved clinched joint. With the increase of the shearing force, the damage developed gradually from the upper sheet material. Then, subsequent crack propagation lead to neck failure and damage. With one damaged part of the neck, the whole bottom of the upper sheet was separated from the other sheet. Then mixed failure mode was generated.

**Figure 11:**
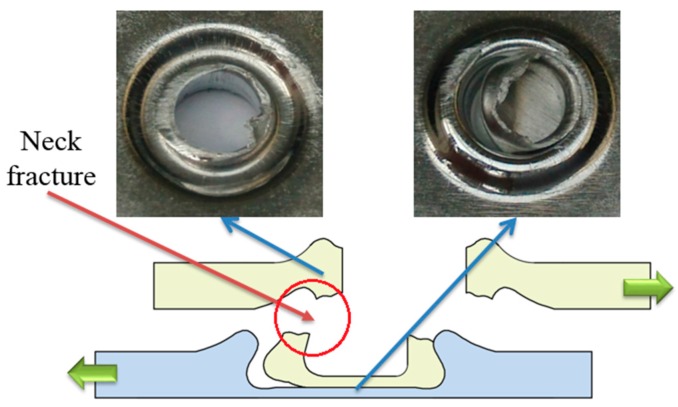
Neck fracture mode in the shearing test.

As shown in Figure 11, the improved clinched joints with forming forces of 70, 80, and 90 kN were failed as the neck fracture mode in the shearing strength test. The mechanical interlock was enhanced by increasing the forming force. The shearing force which the interlock can bear was larger than that which the neck can bear. Thus, the whole neck was fractured in the shearing strength test, which resulted in neck fracture. The bottom part of upper sheet was still kept in the cavity of lower sheet because of the increased mechanical interlock.

**Figure 12:**
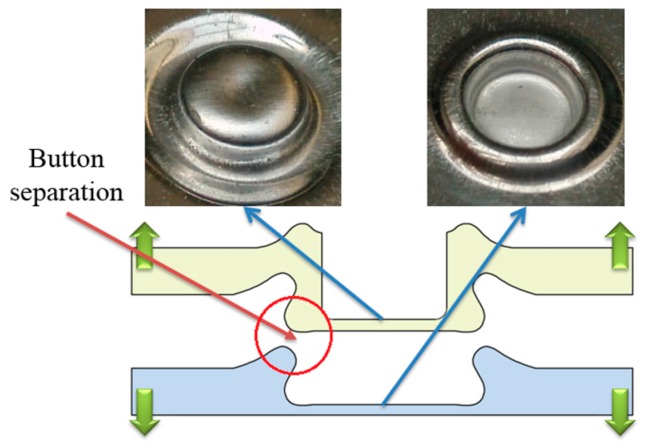
Button separation mode in the tensile test.

In the tensile strength test, button separation mode, mixed failure mode and neck fracture mode were observed in this study. As shown in Figure 12, the clinched joints with forming forces of 60 and 70 kN were failed in the button separation mode. This means the tensile force which the interlock can bear is smaller than that which the neck can bear. The bottom part of upper sheet was separated from the other sheet because of the small mechanical interlock. There was no fracture generated on the joint neck.

**Figure 13:**
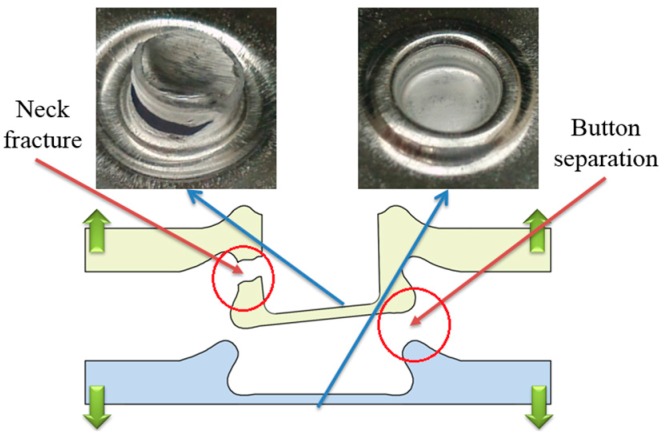
Mixed failure mode in the tensile test.

Figure 13 shows the improved clinched joint formed by a force of 80 kN failed in the mixed failure mode. One half of the neck was fractured, and the other half of the neck was only deformed. In fact, there is a little tool eccentricity between the punch and the blank holder, which results in the uneven interlock and neck along the joint. One part of the neck may have a larger neck thickness and smaller interlock than another part of the neck. For one part of the joint, the tensile force which the interlock can bear is smaller than that which the neck can bear, and for another part of the joint, the tensile force which the interlock can bear is larger than that which the neck can bear. Then one half part of the neck was fractured because of the larger interlock, and another half part of the neck was only deformed with no fracture because of the larger neck thickness. The uneven interlock and neck along the neck resulted in the mixed failure mode. 

**Figure 14:**
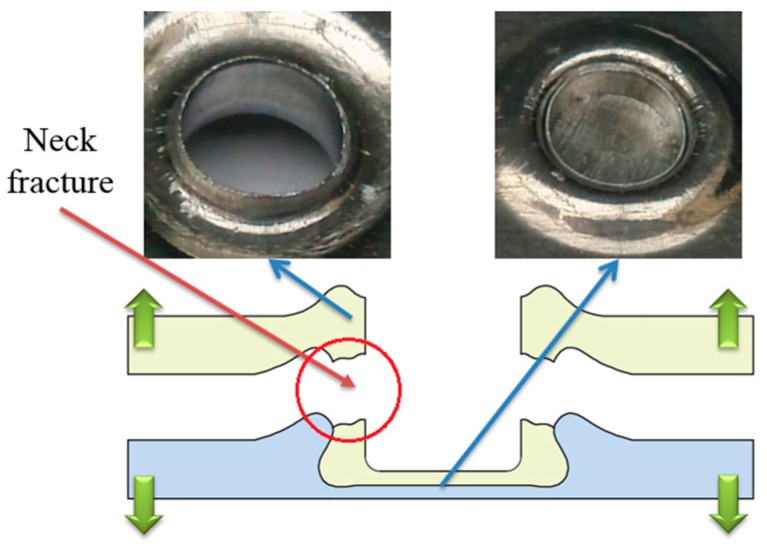
Neck fracture mode in the tensile test.

As shown in Figure 14, the improved clinched joint formed by a force of 90 kN failed in the neck fracture mode. In the tensile test, the tensile force was mainly exerted on the neck. If the tensile stress was increased to exceed the fracture stress of Al1060, the neck part of the improved clinched joint would be fractured. With the increase of the tensile force, the damage developed gradually from the upper sheet material. Then subsequent crack propagation leaded to neck failure and damage.

### 4.4. Static Shearing and Tensile Strength

**Figure 15:**
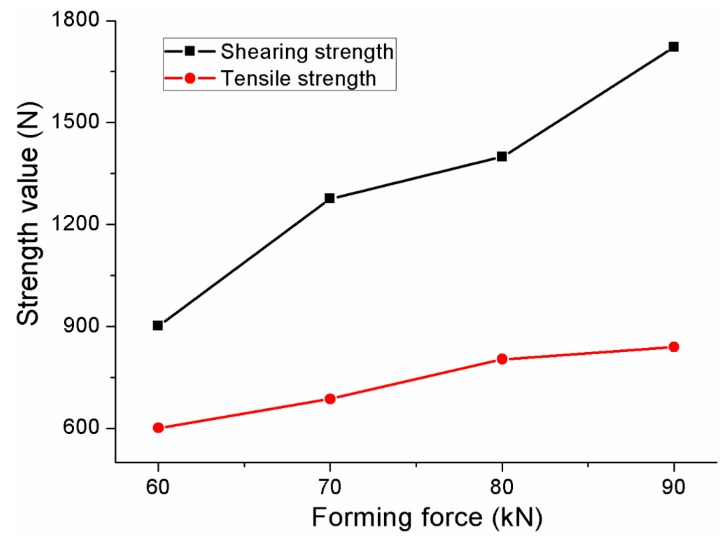
Shearing and tensile strengths of the improved clinched joints.

The static shearing and tensile strengths of the improved clinched joints with forming forces between 60 and 90 kN are shown in Figure 15. The improved clinched joint with a forming force of 60 kN has the lowest shearing and tensile strength, while the improved clinched joint with a forming force of 90 kN has the highest shearing and tensile strength. The shearing strength of improved clinched joint with a forming force of 90 kN was 91.1% higher than that with a forming force of 60 kN, and the tensile strength of the improved clinched joint with a forming force of 90 kN was 39.6% higher than that with a forming force of 60 kN. The shearing and tensile strength could be increased markedly by increasing the forming force.

**Figure 16:**
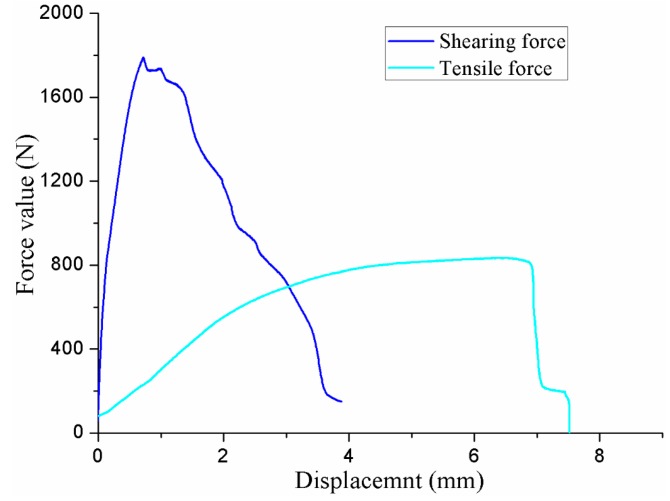
Shearing and tensile force-displacement curves of the improved clinched joints.

The improved clinched joint with a forming force of 90 kN had the highest shearing strength and tensile strength in this study. The shearing and tensile force-displacement curves with a forming force of 90 kN are drawn in Figure 16. The shearing force curve is on the rise with the increase of the shearing displacement before the maximum force value, while the shearing force curve is decreased gradually with the increase of the shearing displacement after the maximum force value. Before the maximum force value, the tensile force curve was gradually on the rise with the increase of the tensile displacement. After the maximum force value, the tensile force curve dropped suddenly. 

## 5. Conclusions

In the present study, an improved clinched joint was investigated by experimental methods. The improved clinching tools with a flat anvil were used to produce the improved clinched joints using different forming forces. Shearing strength, tensile strength, material flow, main geometrical parameters, and failure mode were investigated. The main conclusions of this study can be drawn as follows:

(1) Mechanical interlock was generated to hook the sheets together by plastic deformation for the improved clinched joints with forming forces between 60 and 90 kN.
(2) The material flowing radially and upward could be gathered at the interlock and neck thickness. The increase of the forming force will lead to the increase of the interlock and neck thickness.
(3) Mixed failure mode and neck fracture mode were observed in the shearing strength test, while button separation mode, mixed failure mode and neck fracture mode were observed in the tensile strength test.
(4) The tensile strength and shearing strength can be increased by increasing the forming force. The improved clinched joint produced by a force of 90 kN had highest shearing strength and tensile strength in this study.
